# Maxillary and Frontal Bone Simultaneously Involved in Brown Tumor due to Secondary Hyperparathyroidism in a Hemodialysis Patient

**DOI:** 10.1155/2013/909150

**Published:** 2013-08-19

**Authors:** Suheil Artul, Abdalla Bowirrat, Mustafa Yassin, Zaher Armaly

**Affiliations:** ^1^EMMS Nazareth, The Nazareth Hospital, Faculty of Medicine Bar-Ilan, Galilee University, 16100 Nazareth, Israel; ^2^The Nazareth Hospital, 16100 Nazareth, Israel

## Abstract

Brown tumors are rare focal giant cell lesions of the bone caused by primary hyperparathyroidism (HPT). Brown tumor was reported in 1891; it presents as the end-stage findings of HPT. Common involvements are skull and pelvic girdle. We describe a case of 46-year-old female hemodialysis patient, with secondary HPT in whom multiple masses lesions of the left maxillary sinus and frontal bone were radiologically suspected to be brown tumor. This unusual manifestation of secondary HPT can be expected to occur with increased longevity of patients with renal failure and illustrates the need to include brown tumor in the differential diagnosis.

## 1. The Case Report 

A 46-year-old woman with end-stage renal failure from hypertensive nephrosclerosis that had been treated by hemodialysis for 11 year presented to the Emergency Room at EMMS Nazareth Hospital with vomiting after head injury. Immediate investigations including laboratory testing and head imaging (noncontrast computed tomography—CT scan) were performed to exclude intracranial hemorrhage. The patient recovered from vomiting in the next day, and she was asymptomatic regarding the lesions in the skull.

Our patient's past medical history reveals that she was in relatively good health until the age of 26 when she started to complain of severe and colicky pain, radiating from the back, down the flank, and into the groin not improved by changes in position accompanied by nausea and vomiting. In April 1993, clinical diagnosis and evaluations revealed ESRD due to nephrolithiasis, and chronic hemodialysis treatment was started. In 2004, she received a kidney transplant from a cadaveric donor. In April 2009, she developed chronic allograft rejection, and hemodialysis treatment was restarted.

**Figure 1:**
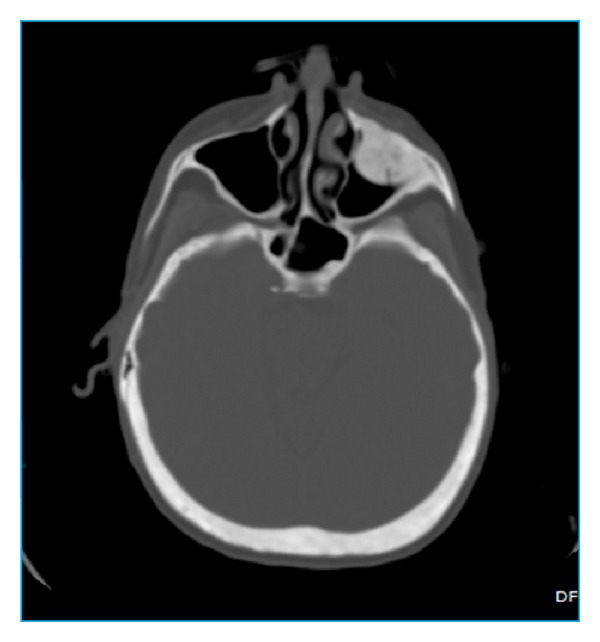
Axial noncontrast head scan at the level of maxillary sinus showing expansive sclerotic mass lesion within the left maxillary sinus without periosteal reaction.

**Figure 2:**
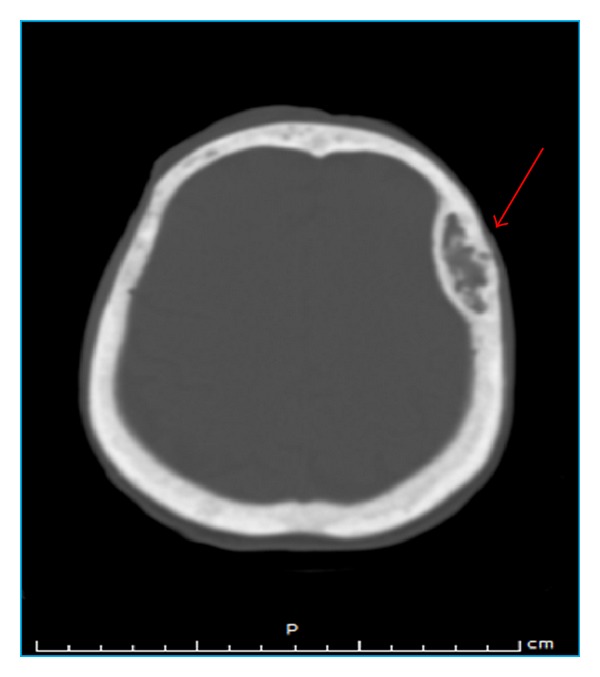
Noncontrast head scan showing expansible lytic “lacelike” lesion (4 × 1.3 cm) of left frontal bone (red arrow) with sclerotic border and amorphous calcifications or “unformed” deposit of calcium and without periosteal reaction. Note also the general sclerosis and the lack of normal appearance of diploica of skull.

In December 2012, the patient was referred to the emergency room at our hospital after head injury. Extended investigations excluded intracranial hemorrhage and fractures; on the other hand, other accidental findings were observed which include expansive sclerotic mass lesion within the left maxillary sinus (2.2 cm in diameter) without periosteal reaction (Figure 1) and an expansible lytic “lacelike” lesion (4 × 1.3 cm) of left frontal bone with sclerotic border and amorphous calcifications or “unformed” deposit of calcium and without periosteal reaction (Figure 2), and all the bones of the skull showed general sclerosis and lack of differentiation of the normal appearance of the diploica in flat table bone of the skull.

The lytic type of this entity mimics metastasis, and therefore diagnosis requires a systemic investigation and good radiological knowledge of this entity for lesion differentiation.

The laboratory results were as follows. Her serum calcium level was 8.5 mg/dL (normal range, 8.5 to 10.5 mg/dL). Her serum phosphorus level was 4.1 mg/dL (normal range, 2.0 to 5.0 mg/dL). Her alkaline phosphate concentration was 406 UI/l (normal range, 37 to 147 IU/lt), and her parathyroid hormone level was 1282 pg/mL (accepted range for stage-V chronic kidney disease, 250–300 pg/mL), not to mention the fact that the patient refused to undergo surgical treatment. Other hormone levels were within their normal ranges. Renal functional tests showed a blood urea level of 126 mg/dL (normal range, 15 to 50 mg/dL), a creatine level of 6.68 mg/dL, a uric acid level of 5.2 mg/dL (normal range, 2.0 to 8.6 mg/dL), and a potassium level of 5.13 mEq/lt (normal range, 3.2 to 5.2 mEq/lt). The patient was treated for secondary hyperparathyroidism with vitamin D, calcium, phosphate binders, and reduced phosphate intake.

Histological examination of the frontal bone lesion revealed giant bone cells and osteitis fibrosa cystica. Considering the clinical, radiographic, histopathological, and blood investigation findings, we reached a final consensus that our patient suffers from brown tumor.

## 2. Discussion

Brown tumor is a rare benign reactive, destructive process of bone caused by a clinical consequence of untreated severe primary, secondary, or tertiary hyperparathyroidism (HPT). A “brown tumor” is not a true neoplasm but a term commonly used for osteitis fibrosa cystica [1]. In fact, brown tumors are one of the complex pathological expressions of osteitis fibrosa cystica. Osteitis fibrosa cystica is a late manifestation of severe hyperparathyroidism (HPT). Other findings of osteitis fibrosa cystica include generalized demineralization of bone, “salt and pepper” appearance of the skull, bone cysts, and brown tumors [2].

The term “brown tumor” is derived from the characteristic appearance of brownish material within the cystic lesion, which is caused by the vascularity, haemorrhage, and deposits of haemosiderin [3].

It is well known that brown tumors, or osteoclastomas, are caused by localized, rapid, osteoclastic removal of bone secondary to the direct effects of PTH on the bone; it is actually a giant cell lesion and often appears as an expansile osteolytic lesion of the bone. They represent localized bony accumulations of fibrous tissue and giant cells that can occur in patients with primary, secondary, and tertiary HPT. Common involvements are long bones, ribs, clavicle, hand, skull, pelvic girdle, and mandible. While the mandible is the most frequently involved bone in the head and neck region, atypical involvement of the cranium in the area of the maxillary sinus and the frontal bone simultaneously, as presented in our case, is rare and had never been reported before in the literature [4].

In 1891, Von Reckling Housen recognized the clinical impact of HPT and described osteitis fibrosa cystica as the pathognomonic bone lesion of this entity. In 1962, Mandle operated on a parathyroid tumor in a patient with hypercalcemia and radiological changes of osteitis fibrosa cystica and demonstrated postoperative regression of the bone disease and biochemical abnormalities.

Incidence of primary HPT has been reported as approximately 5 per 10,000 populations per year. Among patients over 60 years of age the frequency is even higher, approaching nearly 1 per 1000 in men and twice that in women [5].

HPT is a condition caused by high circulatory levels of parathyroid hormone. The various types of HPT manifest different laboratory findings. In primary HPT, the serum calcium level is usually elevated, with low or normal serum phosphate level. Patients with secondary HPT, on the other hand, usually present with hypocalcaemia and hyperphosphataemia. These findings are variable in tertiary HPT. In addition, HPT is also classified according to severity into 4 types: primary due to hyperplasia, benign, or malignant neoplasia of one or more of the parathyroid glands; secondary, when the parathyroid gland is stimulated to produce increased amount of hormone to correct abnormally low serum calcium levels in different physiologic or pathologic conditions resulting in parathyroid hyperplasia; Tertiary, when long-standing secondary hyperplasia becomes autonomous in spite of correction of the underlying stimulant; and a fourth type is ectopic HPT observed in patients with malignant disease [6].

A tight relationship between chronic secondary HPT, and chronic renal failure (CRF) is well known. Most patients with CRF present some degree of secondary HPT and modifications of parathyroid glands begin early during the development of the renal disease [6]. Secondary HPT is a frequent complication of CRF as a consequence of renal osteodystrophy. CRF results in decreased phosphate excretion, hyperphosphatemia, and hypocalcemia. These factors lead to stimulation of the parathyroid glands. Indeed, a spectrum of skeletal abnormalities seen in CRF are as follows: Osteitis fibrosa cystica, characterized by increased osteoclast and osteoblasts activity, peritrabecular fibrosis, and high serum level of PTH (usually higher than 350–500 pg/mL); adynamic bone disease, characterized by low or absent bone formation due to low osteoblast-bone formation and osteoclast-bone resorbing activities accompanied by low serum levels of PTH; and osteomalacia, osteopenia or osteoporosis, and mixed renal osteodystrophy.

The incidence of skeletal brown tumors in chronic renal failure (CRF) ranges from 1.5 to 13% but is expected to decrease with improvements in the medical care of these patients [6]. This unusual complication of secondary HPT is more commonly seen in young female patients, with prolonged treatment period of hemodialysis, as was the case of our patient [7].

Histologically, brown tumors are nonencapsulated and are characterized by abundant stroma consisting of fibrous connective tissue, with important proliferation of fibroblasts and several multinucleated osteoclast-like giant cells containing variable numbers of nuclei. Calcified material and areas with extravasations of red blood cell and hemosiderin in histiocyte can be found [8]. The cystic spaces are filled up by clusters of giant cells, hemosiderin-laden macrophages, and plump fibroblasts. Cystic formation may develop as a result of bleeding and tissue degeneration. Giant cell masses or brown tumors may result from these changes and are usually seen as focal bone lesions. Giant cells are similar to the other giant cell lesions (true giant cell tumor, reparative giant cell granuloma, cherubism, and aneurysmal bone cyst).

Histological features alone cannot establish a certain diagnosis of a brown tumor. Indeed, on clinical examination and using only routine panoramic radiography, the lesions may resemble osteosarcoma, bone metastases of a carcinoma, multiple myeloma, Langerhan's cell histiocytosis, Paget's disease, osteomyelitis, or osteonecrosis secondary to bisphosphonate therapy. The differential diagnosis is based on the clinical history and widespread skeletal involvement; pathological fractures and renal stones may suggest the presence of primary HPT. The diagnosis of HPT should be established by determination of the serum calcium, phosphorus, ALP, and PTH level rather than by histological examination of focal lesion alone. The main challenge in diagnosing brown tumor (as was the case of our patient) is that it can be seen in normocalcemic patients as well [9].

Radiological examination: on CT scan, brown tumors appear as a lytic or sclerotic lesion without periosteal reaction. The lesion may be hyperdense or heterogeneous and well-circumscribed or expansile lucent lesion with a rim of calcification and remodeling of surrounding bone. After injection of contrast medium, brown tumors appear as heterogeneously enhancing masses [10]. There is no periosteal reaction or soft tissue invasion [10]. The appearance on CT is challenging and not always specific and mimics metastasis [10].

The most significant point about the case described here is the simultaneous appearance of brown tumors in the left maxillary sinus “sclerotic pattern” and in the left frontal bone “lytic pattern” with amorphous calcifications and the general sclerosis of the cranial bones.

In conclusion this case should attract the attention of general practitioner dentists, oral and maxillofacial surgeons, endocrinologists, and especially radiologists whose consultation is essential in including this entity in the differential diagnosis to avoid unnecessary surgical removal. Accurate diagnosis enabled the proper treatment to be carried out, avoiding unnecessary harm to the patient.
